# The prevalence and correlates of anxiety and depression amongst essential workers during the COVID-19 lockdown in Ekiti State, Nigeria

**DOI:** 10.4102/sajpsychiatry.v28i0.1610

**Published:** 2022-02-24

**Authors:** Joshua Falade, Adedayo H. Oyebanji, Abayomi M. Oshatimi, Adefunke O. Babatola, Adefolurin Orekoya, Benjamin A. Eegunranti, Olusola O. Falade

**Affiliations:** 1 Department of Mental Health, Afe Babalola University, Ado-Ekiti, Ekiti State, Nigeria; 2 Department of Paediatrics, Afe Babalola University, Ado-Ekiti, Ekiti State, Nigeria; 3 University Health Center, Federal University Oye, Ekiti, Ekiti State, Nigeria; 4 Department of Paediatrics, Ekiti State University, Ado Ekiti, Ekiti State, Nigeria; 5 Department of Psychiatry, Ladoke Akintola University of Technology, Ogbomoso, Oyo State, Nigeria; 6 Osun State School of Nursing, Asubiaro, Osogbo, Osun State, Nigeria

**Keywords:** essential workers, health workers, non-health workers, anxiety, depression, COVID-19, pandemic, Nigeria

## Abstract

**Background:**

Essential workers are imperative in the fight against the coronavirus disease (COVID-19) pandemic.

**Aim:**

To assess the prevalence and factors associated with anxiety and depression among essential workers during the COVID-19 pandemic and lockdown.

**Setting:**

This study was set in Ekiti State, Nigeria.

**Method:**

This was a cross-sectional study involving essential workers in Ekiti State Nigeria, during the COVID-19 pandemic and lockdown. A total of 588 essential workers were sampled. Online socio-demographic variables and the Hospital Anxiety and Depression Scale, a 14 item self-reported questionnaire were used.

**Results:**

The prevalence of anxiety and depression among the respondents was 93.4% (CI = 91.2–95.2) and 64.3% (CI = 60.4–68.4) respectively. Among the health workers, the prevalence of anxiety and depression were 96.5% (CI =94.8–98.1) and 66.5% (CI = 60.5–69.8) respectively while the prevalence of anxiety and depressive symptoms among non- health workers were 84.6% (CI = 78.7–90.1) and 61.5% (CI = 54.2–69.4) respectively. The odds ratio (OR) of depressive symptoms was increased among, respondents who were not satisfied with the support they received from the government during the pandemic (AOR = 2.071, CI = 1.350–2.213), respondents that were 35 years and younger (AOR = 1.512, CI = 1.033–2.213) and reduced amongst Christians (AOR = 0.501, CI = 0.286–0.879). The odd of anxiety was increased among health workers compared to non-health workers (AOR = 3.700, CI = 1.744–7.851) and the odds of anxiety was reduced among respondents with previous history of mental illness (AOR = 0.215, CI = 0.049–0.943).

**Conclusion:**

Anxiety and depressive symptoms were common mental illness among essential workers working during the COVID-19 lockdown, therefore their mental health should be adequately considered to sustain the fight against the virus.

## Background

Coronavirus disease 2019 (COVID-19), an extremely contagious and serious acute respiratory syndrome (SARS) caused by a novel coronavirus (SARS-CoV-2), began in Wuhan, China, in December 2019, and it was later declared a pandemic on 11 March 2020 by the World Health Organization (WHO).^1^ Globally, more mortality by COVID-19 virus has been reported than by the previous SARS and the Middle East respiratory syndrome (MERS) combined.^2,3^ The pandemic has led to quarantining of whole communities, premature and prolonged closure of schools as well as physical (social) distancing and isolation. All these have presently altered everyday life.^4^

The public psychological response to a pandemic goes a long way to determine the outcome of the disease during and after any infectious disease outbreak.^5,6^ Generally, an exaggerated rise of fear and anxiety amongst people because of vagueness of the disease together with essential but socially disruptive measures like lockdowns and quarantines can lead to significant mental health disturbances such as post-traumatic stress disorder (PTSD), depression, anxiety, panic disorders and behavioural disorders.^7^

Extraordinary amounts of pressure on healthcare workers have been documented during previous epidemics (Ebola and SARS).^8^ Increased workload, physical exhaustion, inadequate personal protective equipment (PPE), the transmission of nosocomial infection and the need to make ethically difficult decisions like the rationing of care were documented risk factors for mental illness amongst healthcare workers during the COVID-19 pandemic.^9^ Essential workers (healthcare and non-healthcare workers) may be specifically susceptible to experiencing psychological trauma during a pandemic period.^10^

A study in China amongst healthcare workers reported that the prevalence of symptoms of anxiety, depression, insomnia and the overall psychological problems in healthcare workers during the COVID-19 pandemic were 46.04%, 44.37%, 28.75% and 56.59%, respectively.^11^ In Singapore during the same COVID-19 period, a study amongst health workers found the prevalence of anxiety, depression, stress and PTSD to be 14.50% 8.90%, 6.60%, and 7.70%, respectively.^6^ A systematic review amongst health workers reported a pooled prevalence of 23.20% and 22.80% for anxiety and depression, respectively.^12^

A Chinese nationwide study amongst medical personnel during the COVID-19 pandemic reported that half of the responders reported at least mild depression and one-third reported insomnia,^13^ whereas 14% of physicians and nearly 16% of nurses described moderate or severe depressive symptoms.^13^ The researchers found that the female frontline workers were direct and independent risk factors for developing abnormal stress symptoms.^13^

Maintaining a sustained fight against COVID-19 infection requires that healthcare professionals have sustained physical and mental health.^4^

A complete understanding of the emotional burden amongst essential workers in Nigeria and globally during this period is important for providing psychological support, establishing and improving mental healthcare support services. Since COVID-19 is a novel viral infection, there is a dearth of study and data on the psychological sequel of the infection on essential workers globally, and particularly in Nigeria. This study, therefore, aimed to assess the prevalence and factors associated with anxiety and depression amongst essential workers during the COVID-19 pandemic and lockdown in Ekiti State, Nigeria.

## Materials and method

### Study settings, participants and design

This was a cross-sectional descriptive study involving essential workers working in Ekiti State during the COVID-19 lockdown. Ekiti State is one of the Yoruba states in Nigeria, mainly the highland zone, rising above 250 m above sea level. The state is situated entirely within the tropics located between longitudes 40°51′ and 50°451′ East of the Greenwich meridian and latitudes 70°151′ and 80°51′ north of the Equator.

Study participants were essential workers, mainly professionals, who were working during the COVID-19 lockdown in Ekiti State, Nigeria, and these include healthcare workers, security personnel, financial sector workers, COVID-19 monitoring committees, senior political office holders and senior civil servants.

The total list and WhatsApp numbers of the essential workers going to work during the COVID-19 lockdown were collected from their respective heads of units. The online questionnaires were sent to those whose phone numbers were WhatsApp-enabled and responses were sent back to the investigators via the number as a result of the ban on interstate travel and social distancing during the lockdown. A few of the respondents were contacted to fill the questionnaire properly. The study occurred from March to July 2020.

### Sample size

The minimum sample size was calculated using the formula for prevalence studies.^14,15^


$$N=\frac{Z^{2}pq}{d^{2}}$$

where *N* is the desired sample size if the population is more than 10 000; *Z* is the standard normal deviate usually set at 1.96 corresponding to 95% confidence interval (CI); *p* is the proportion in the target population estimated to have particular characteristics; *q* = 1 – *p*; and *d* is the degree of accuracy desired set at 0.05

The prevalence of 50.0% is used which represents the prevalence of an unknown character in a population.^16^ = 384

### Study instrument

Data were collected through online questionnaires sent to the respondents. The questionnaires comprised the following:

1. The Socio-Demographic Schedule: This section contained information on the socio-demographic profiles of respondents.
2. Hospital Anxiety and Depression Scale (HADS):

Zigmond and Snaith developed the HADS.^17^ It is a self-report, efficient instrument to the determination of the presence and severity of both depression and anxiety while giving the scores for each item.^18^ The instrument has been used in Nigeria and the sensitivity for anxiety and depression subscales ranged from 85.0% to 92.9% and 89.5% to 92.1%, respectively, while the specificity for the anxiety and depression ranged from 86.5% in the gynaecology clinic to 90.6% in the community sample, and 86.6% in the medical and surgical wards to 91.1% in community sample, respectively.^19,20^ It has also been validated for different settings.^21^

The instrument consists of 14 items (seven questions each for anxiety and depression). The responses are scored on a Likert scale. Each question has four responses ranging from not at all to very often. Zero marks are allocated to ‘not at all’ while 3 is allocated to ‘very often’. The minimum and maximum obtainable scores are 0 and 21, respectively, each for anxiety and depression. Scores less than 7 are considered as normal; 8–10 as borderline abnormal (borderline cases) while 11–21 as abnormal case. For this study, 0–7 is considered as negative while borderline abnormal and abnormal were regrouped as positive for both anxiety and depressive symptoms.

### Data analysis

The Statistical Package for Social Sciences (SPSS version 21) was used for data analysis. The socio-demographic details of respondents were reported using descriptive statistics such as proportions and frequencies. Chi-square tests were used to determine the relationship between socio-demographic details, and anxiety and depressive symptoms. Multivariate statistical techniques such as binary logistic regression were employed to identify the factors that were significantly associated with anxiety and depressive symptoms amongst the respondents. The CI was set at 95% and all tests were two-tailed. Statistical significance was considered at a *p*-value of less than 0.05.

### Ethical considerations

The Research Ethics Committee of the Ekiti State University Ado Ekiti gave ethical approval and a letter of permission was collected from various institutions to conduct the study. Informed consent was given by respondents. Protocol number: EKSUTH/A67/2020/05/003.

## Results

### Socio-demographic characteristics of the respondents

Five hundred and eighty-eight questionnaires were completed and returned out of a total of 700 questionnaires that were sent to the WhatsApp numbers of the essential workers at work during the COVID-19 lockdown giving a response rate of 84%.

Table 1 shows the socio-demographic characteristics of respondents. Most (56.1%) of the respondents were ≤ 35 years; a higher proportion of the respondents were male (67.5%), Christians (85.7%), health workers (73.5%), married (71.9%) and from Yoruba ethnicity (82.5%). One hundred and thirty-six (23.1%) had a previous history of mental illness while 6.1% of the respondents had a history of mental illness. A significant proportion of the respondents (74.5%) receives two hundred and fifty thousand naira or or less per month, the majority (72.4%) were not satisfied with the support they received from the government during this pandemic, while 66.8% of the respondents were not satisfied with the support they received from their organisation during this pandemic.

**TABLE 1:** Socio-demographic characteristics of the respondents.

Variable	Frequency (*n* = 588)	Percentage (%)
**Age**		
≤ 35 years	330	56.1
> 36 years	258	43.9
**Gender**		
Female	191	32.5
Male	397	67.5
**Religion**		
Christianity	504	85.7
Islam	84	14.3
**Occupation**		
Health worker	432	73.5
Non-health worker	156	26.5
**Marital status**		
Single	156	26.5
Married	423	71.9
Separated	9	1.5
**Family type**		
Monogamous	546	92.9
Polygamous	42	7.1
**Tribe**		
Yoruba	485	82.5
Hausa	30	5.1
Igbo	73	12.4
**Previous history of mental illness**		
Yes	136	23.1
No	452	76.9
**Family history of psychiatric disorder**		
Yes	36	6.1
No	552	93.9
**Salary (Nigerian Naira)**		
≤ 250 000	438	74.5
> 250 000	150	25.5
**Satisfaction with government**		
Not satisfied	426	72.4
Satisfied	162	27.6
**Satisfaction with organisation**		
Not satisfied	474	80.6
Satisfied	114	19.6

### Prevalence of anxiety and depressive symptoms amongst the respondents

The prevalence of anxiety and depressive symptoms amongst essential workers at work in Ekiti State during the COVID-19 lockdown was 93.4% (CI = 91.2–95.2) and 64.3% (CI = 60.4–68.4) respectively. Amongst the health workers, the prevalence of anxiety and depressive symptoms was 96.5% (CI = 94.8–98.1) and 66.5% (60.5–69.8), respectively, while the prevalence of anxiety and depressive symptoms amongst non-healthcare essential worker was 84.6% (CI = 78.7–90.1) and 61.5% (CI = 54.2–69.4).

### Association of depressive symptoms with socio-demographic characteristics of the respondents

Table 2 shows the association between depressive symptoms and socio-demographic characteristics of the respondents based on the comparison of 210 subjects without depressive symptoms and 378 subjects with depressive symptoms. More respondents, ≤ 35 years, had depressive symptoms compared to those greater than 35 years and the difference was statistically significant (*p* ˂ 0.001). Also, there was an association between religion and depressive symptoms (*p* = 0.027). Additionally, more respondents who were single (75%) had depressive symptoms more than others, with a statistically significant difference (*p* = 0.001). Similarly, more individuals from the Yorubas’ tribe had depressive symptoms than other tribes, with a statistically significant difference (*p* = 0.009). More respondents with no family history of psychiatric disorder (65.8%) had depressive symptoms than those with family history (41.7%), with a statistically significant difference (*p* = 0.003). Furthermore, more respondents not satisfied with the support they received from the government during the pandemic had depressive symptoms, and the difference was statistically significant (*p* ˂ 0001). More respondents who were not satisfied with the support they received from their organisation had depressive symptoms compared with those who were satisfied (*p* = 0.021).

**TABLE 2:** Association of socio-demographic characteristics of the respondents with depression.

Variable	Depression				*p*
	Negative		Positive		
	*n*	%	*n*	%	
**Age**					
≤ 35 years	96	29.1	234	70.9	< 0.001
> 36 years	114	44.2	144	55.8	-
**Gender**					
Female	65	34.0	126	66.0	0.555
Male	145	36.5	252	63.5	-
**Religion**					
Christianity	189	37.5	315	62.5	**0.027**
Islam (Muslim)	21	25.0	63	75.0	-
**Occupation**					
Health worker	150	34.7	282	65.3	0.403
Non-health worker	60	38.5	96	61.5	-
**Marital status**					
Single	39	25.0	117	75.0	**0.001**
Married	165	39.0	258	61.0	-
Separated	6	66.7	3	33.3	-
**Family type**					
Monogamous	201	36.8	345	63.2	0.445
Polygamous	9	21.4	33	78.6	-
**Tribe**					
Yoruba	182	37.5	303	62.5	**0.009**
Hausa	3	10.0	27	90.0	-
Igbo	25	34.2	48	65.8	-
**Previous history of mental illness**					
Yes	53	39.0	83	61.0	0.366
No	157	34.7	295	65.3	-
**Family history of psychiatric disorder**					
Yes	21	58.3	15	41.7	**0.003**
No	189	34.2	363	65.8	-
**Salary**					
≤ 250 000	150	34.2	288	65.8	0.204
> 250 000	60	40.0	90	60.0	-
**Satisfaction from government**					
Not satisfied	126	29.6	300	70.4	**< 0.001**
Satisfied	84	1.9	78	48.1	-
**Satisfaction from organisation**					
Not satisfied	153	32.3	321	67.7	**0.001**
Satisfied	57	50	57	50.0	-

### Association of anxiety with socio-demographic characteristics of the respondents

Table 3 shows the comparison of the socio-demographic characteristics between 39 subjects without anxiety and 549 subjects with anxiety. A significant proportion of respondents who were health workers had anxiety disorder more than non-health workers. The difference was statistically significant (*p* ˂ 0.001). Also married people (94.3%) had anxiety more than those who were single and divorced (94.2% and 33.3%, with a statistically significant difference (*p* ˂ 0.001). Additionally, respondents with no history of mental illness (93.8%) had anxiety-related symptoms more than respondents with a history of mental illness (75%). The difference was also statistically significant (*p* = 0.010). Furthermore, those who earn less than two hundred and fifty thousand naira had anxiety more than respondents who earn more. The difference was significant (*p* = 0.008). Also, more respondents who were not satisfied with support from their organisation had anxiety compared with those who were satisfied. The difference was statistically significant (*p* = 0.002).

**TABLE 3:** Association of socio-demographic characteristics of the respondents with anxiety.

Variable	Anxiety disorder				*p*
	Negative		Positive		
	*n*	%	*n*	%	
**Age**					
≤ 35 years	27	8.2	303	91.8	0.088
> 36 years	12	4.7	246	95.3	-
**Gender**					
Female	12	6.8	179	93.7	0.813
Male	27	6.8	370	93.2	-
**Religion**					
Christianity	36	7.1	315	92.9	0.223
Islam	3	3.6	81	96.4	-
**Occupation**					
Health worker	15	3.5	417	96.5	< 0.001
Non-health worker	24	15.4	132	84.6	-
**Marital status**					
Single	9	5.8	147	94.2	**< 0.001**
Married	24	5.7	399	94.3	-
Separated	6	66.7	3	33.3	-
**Family type**					
Monogamous	39	7.1	507	92.9	0.073
Polygamous	0	0.0	42	100	-
**Tribe**					
Yoruba	31	6.4	303	93.6	0.112
Hausa	0	0.0	30	100	-
Igbo	8	11.0	65	89.0	-
**Previous history of mental illness**					
Yes	3	25.0	9	75.0	**0.010**
No	36	6.3	540	93.8	-
**Family history of psychiatric disorder**					
Yes	3	8.3	33	91.7	0.672
No	36	6.5	516	93.5	-
**Salary**					
≤ 250 000	36	8.2	402	91.8	**0.008**
> 250 000	3	2.0	147	98.0	-
**Satisfaction from government**					
Not satisfied	33	7.7	393	92.3	0.078
Satisfied	6	3.7	156	96.3	-
**Satisfaction from organisation**					
Not satisfied	24	5.1	451	94.9	**0.002**
Satisfied	15	13.22	99	86.8	-

### The socio-demographic variables independently associated with depressive symptoms by logistic regression analysis

Table 4 shows the results of logistic regression analysis with a 95% CI using a stepwise method to explore the factors independently associated with depressive symptoms. The socio-demographic variables were entered as independent variables and depressive symptoms were entered as dependent variables. The result revealed that respondents who were not satisfied with the support they received from the government during the pandemic had increased odds of depressive symptoms compared with the satisfied ones (odds ratio [OR] = 2.333, CI = 1.447–3.445). Respondents who were 35 years and below were more likely to have depressive symptoms than respondents who were older than 35 years (adjusted odds ratio [AOR] = 1.512, CI = 1.033–2.213); finally, the respondents who were Christians were less likely to have depressive symptoms than those who were Muslims (AOR = 0.501, CI = 0.286–0.879).

**TABLE 4:** The socio-demographic variables independently associated with depression by logistic regression.

Variable	Adjusted odds ratio	*p*	95% Cl for EXP(B)	
			Lower	Higher
**Ethnicity**				
Igbo and Hausa (ref.)	0.774	0.312	0.470	1.273
Yoruba				
**Marital status**				
Married (ref.)	1.229	0.335	0.794	1.903
Single and separated				
**Religion**				
Islam (ref.)	0.501	0.016	0.286	0.879
Christianity				
**Satisfied with organisation support**				
Satisfied (ref.)	0.799	0.281	0.532	1.201
Not satisfied				
**Family history of psychiatric disorder**				
No (ref.)	0.532	0.080	0.255	1.08
Yes				
**Satisfied with government support**				
Satisfied (ref.)	2.071	< 0.001	1.350	3.172
Not satisfied				
**Age**				
36 and above (ref.)	1.512	**0.0331**	1.033	2.213
≤ 35				

ref., reference point which is the variable to which others are being compared.

### The socio-demographic variables independently associated with anxiety by logistic regression analysis

Table 5 shows the results of logistic regression analysis with a 95% CI using a stepwise method to explore the factors independently associated with an anxiety disorder. The socio-demographic variables were entered as independent variables and anxiety disorder was entered as the dependent variable. The result revealed that the odds of anxiety disorder were less amongst respondents with previous history of mental illness compared with those who had no previous history of mental illness (OR = 0.215, CI = 0.049–0.943). Also, health workers are about 4.0 times more likely to develop anxiety compared to non-health workers (OR = 4.08, CI = 1.997–8.346).

**TABLE 5:** The socio-demographic variables independently associated with anxiety disorder by logistic regression analysis.

Variable	Adjusted odds ratio	*p*	95% Cl for EXP(B)	
			Lower	Higher
**Marital status**				
Married(ref.)	0.574	0.130	0.280	1.178
Single and separated				
**Satisfied with organisation support**				
Satisfied (ref.)	1.310	0.445	0.655	2.619
Not satisfied				
**previous history of mental illness**				
No (ref.)	0.215	**0.042**	0.049	0.943
Yes				
**Salary**				
More than #250 000 (ref.)	0.464	0.233	0.131	1.640
≤#250 000				
**Occupation**				
Non-health worker (ref.)	4.083	**0.001**	1.997	8.346
Health worker				

ref., reference point which is the variable to which others are being compared.

## Discussion

The study determined the prevalence and predictors of anxiety and depressive symptoms amongst essential workers working in Ekiti State during the COVID-19 lockdown. The prevalence of anxiety and depressive symptoms (93.4% and 64.3%) was high amongst essential workers in our study. It is higher than 50.4% for depressive symptoms and 44.6% for anxiety reported amongst health workers in China (13), although our own study included health workers and non-health-related essential workers. Amongst the migrant workers in India during the COVID-19 pandemic, about three-quarters of the participants (73.5%) screened positive for depressive symptoms, and about half of the participants (50%) screened positive for anxiety.^22^ However, a lower prevalence of 34% and 19% for anxiety and depressive symptoms, respectively, was reported amongst health workers in Jordan because the Kingdom of Jordan has documented some of the lowest numbers of cases worldwide because of its prompt and well-founded and stringent response to the outbreak.^23^ The effect of the mental illness amongst essential workers at work during the COVID-19 lockdown cannot be underestimated because it can negatively affect employee performance and productivity, and as such the importance of putting in place measures to mitigate this effect on these workers cannot be overemphasised.

The predictors of depressive symptoms identified amongst the respondents in the study were respondents who were 35 years and less, respondents not satisfied with the government policies, while Christian respondents are less likely to report depressive symptoms.

The role of religious practices is an important concept for psychological well-being.^24^ In this study, respondents who are Christians were less likely to suffer from depressive symptoms. This may be because during the COVID-19 lockdown Christian respondents still had diverse opportunities for the substitution of their physical meetings that were halted earlier by the COVID-19 lockdown. There were various online services introduced and organised by various Christian organisations.^25,26^ For example, Christians were still able to make contributions and give offerings online without much stress, regular church programmes continued online without having to be there physically.^27,28^ Some of these church programmes had been on air even before the lockdown.^29^ Thus, both spirituality for those with intrinsic religiousness and religiosity for those with extrinsic religiousness and both combined were satisfied.^30^ Emphasis on spirituality by religious leaders before this time also helped in this period.

During the COVID-19 lockdown, it is possible that Christians also saw an opportunity for increase in personal devotion time and increase in assumed communion with God afforded by the ample time made available by the lockdown to participate in the various online church programmes.^31,32^

Also, during the lockdown many churches were involved in charity where members of their church and the needy in their communities were the beneficiaries. A study reported that both self-reported religiosity and religious influence were significantly related to depression scores while service/prayer attendance was negatively correlated with depression scores.^33^ The significance and the helpful role of involvement in recognised religious establishments cannot be overemphasised.^30^

Respondents who were 35 years and less were more likely to have depressive symptoms than older respondents. The younger respondents may be the first point of call in the fight against COVID-19. They are the first to be exposed to the infection as health and non-health workers; this may be coupled with lower economic power compared to the older respondents who may be at the higher level in their various disciplines. They may also bear more of the socio-economic instability caused by the COVID-19 pandemic than their older colleagues.

The rapid global spread of COVID-19 has generated a wide range of responses from the government and policy-makers.^31^ The acceptability of the policies by the essential workers and the society at large goes a long way to curb the spread of COVID-19. The Federal Government of Nigeria released intervention fund to help frontline line workers and vulnerable citizens. However, some vulnerable households and frontline workers largely reported that they did not receive any aid from the government, as was initially promised. Non-satisfaction with government support was a risk for depressive symptoms in this study. There are many expectations by the essential workers from the government such as the provision of allowances, PPE, insurance, transportation and training. These unmet expectations may generate worries in the mind of the workers. In addition, inadequate integration of the essential workers into the process of policy formulation and implementation may also lead to a high degree of dissatisfaction.

The predictors of anxiety disorder in this study were having no previous history of mental illness and being a health worker.

Respondents with previous history of mental illness were less likely to have anxiety disorder in this study. This observation is different from the previous studies which described anxiety disorders as recurrent especially during a stressful period.^32,33^ The COVID-19 pandemic has generated fear amongst the general population^1^; therefore, respondents with no history of mental illness may be experiencing this level of anxiety-provoking event for the first time compared to respondents who had the previous history of mental illness, who might have experienced a stressful event and have been attending regular mental health clinics. Respondents who have been regular in the clinic might have learned useful anxiety-reducing strategies and better-coping strategies that might be applicable during the pandemic. Besides, during the lockdown, many mental health experts and some hospitals were offering online consultations and services which might have helped their patients.

Health workers were more likely to have an anxiety disorder; this may be because they are a major stakeholder in this fight against COVID-19. They are exposed to various stressors such as increased workload, physical exhaustion, inadequate PPE, risk of being infected by COVID-19 and the need to make ethically difficult decisions like the rationing of care.^9^ This, therefore, suggests that targeted policies should be formulated such as those that will ensure adequate healthcare funding by government and private health institutions, firm legislation against exploitation of employees, appropriateness of work environment, working tools and work hours.

In conclusion, the COVID-19 lockdown has caused a high level of anxiety with depressive symptoms seen amongst the essential workers going to work in Nigeria and this is in keeping with other studies. The mental health of the essential workers should be adequately considered to improve the overall functionality of the workers.

## Limitation

The study was restricted to the respondents who had smartphones and are literate.
